# How general practitioners used job crafting strategies during the COVID-19 pandemic in Sweden

**DOI:** 10.1080/02813432.2024.2309633

**Published:** 2024-02-05

**Authors:** Helena Månsson Sandberg, Åsa Tjulin, Emma Brulin, Bodil J. Landstad

**Affiliations:** a Department of Health Sciences, Mid Sweden University, Östersund, Sweden; b Unit of Occupational Medicine, Karolinska Institutet, Stockholm, Sweden; c Faculty of Human Sciences, Mid Sweden University, Östersund, Sweden; d Unit of Research, Education and Development, Östersund Hospital, Östersund, Sweden

**Keywords:** Covid-19, general practitioners, job crafting, primary healthcare, work strategies

## Abstract

**Objective:**

General practitioners (GPs) played a crucial role in limiting the impact of the COVID-19 pandemic, and many GPs experienced they did not have the prerequisites to provide adequate care. However, GPs developed approaches that helped them to provide care to patients through various job crafting strategies. The aim of this study is to identify how job crafting strategies were deployed by GPs at the beginning of the COVID-19 pandemic in Sweden and the significance of the strategies on their work situation.

**Design:**

A qualitative design with semi-structured interviews. The data was analysed using qualitative content analysis with job crafting as the conceptual framework for the analysis process.

**Setting:**

Primary healthcare in five healthcare regions in Sweden.

**Subjects:**

Fourteen GPs participated in individual interviews.

**Results:**

In their endeavours to organise and provide care, GPs shaped the task, relational and cognitive boundaries of their work. GPs felt proud about finding new ways of working when given room to manoeuvre. Intensified collaboration between healthcare professionals made GPs more confident in their clinical work. GPs expressed that they consequently felt stronger in their professional role through what they accomplished in the organisation of care.

**Conclusions/Implications:**

The results suggest that the job crafting strategies GPs used were meaningful to them in clinical practice. Knowledge about how GPs’ job crafting strategies were deployed might be useful for healthcare organisations in preparing for future health crises. Taking advantage of GPs’ experiences and strategies is considered important for promoting sustainable working conditions for GPs in the future.

## KEY POINTS

- During the onset of the COVID-19 pandemic in Sweden, general practitioners took immediate action to re-organise their day-to-day work tasks.
- To manage professional uncertainty about how to provide the best possible care, general practitioners sought support from other healthcare peers.
- The professional self-confidence of general practitioners increased through what they accomplished when facing a major health crisis.

## Introduction

During the COVID-19 pandemic, healthcare systems worldwide were under great pressure with crowded wards within hospital-based care [1,2]. The vulnerability of healthcare systems was exposed, not least within primary healthcare [3,4]. General practitioners (GPs), i.e. doctors in primary healthcare centres, were vital in restricting the impact of the pandemic on the general public [5]. Over and above normal provision of care, their role involved identifying risk groups and pinpointing new cases of COVID-19 [5]. Since GPs handled the majority of care required to treat COVID-19, far fewer individuals with the virus needed to be hospitalised [5,6]. However, many GPs experienced they did not have the prerequisites to provide adequate care [7–9]. At the beginning of the pandemic some GPs stated that there was a lack of specific guidelines for how COVID-19 should be managed within primary healthcare [10], while others struggled with changing, confusing and sometimes also conflicting recommendations from different authorities [4,7]. During the pandemic, GPs proved their ability to improvise and apply creative strategies [7,11], and this helped them to provide safe care to patients.

In no time, patient consultations were changed from physical visits to telephone and digital meetings [7,12–15]. GPs continuously shared information and discussed clinical practice in collaboration with other healthcare professionals and medical specialists [7,10,12–14,16]. In addition, some GPs resolved the shortfall in personal protective equipment (PPE) by arranging to get these items from alternative sources, such as industrial suppliers or *via* the internet [7].

Overall, it is clear that GPs took action in response to the COVID-19 pandemic. The theoretical framework of job crafting focuses on the actions taken by the individual to redesign their work in order to enhance meaningfulness, engagement, resilience and well-being at work [17]. Individuals may thus use job crafting strategies to achieve a work situation that they find more appealing [17]. Job crafting can be described as ‘the physical and cognitive changes individuals make in the task or relational boundaries of their work’ and how these strategies revise employees’ work-related identities and work meanings [17]. Three different forms of job crafting can be distinguished: task crafting, relational crafting and cognitive crafting [17]. The first form of job crafting involves shaping work-related task boundaries i.e. how employees change the number, scope or type of job tasks done at work. Relational crafting, the second form, is when employees change the relational boundaries of their work. In other words, they change the quality or amount of interaction with others at work. Through relational crafting at work, employees can decide how frequently they interact with others, and also the quality of those interactions. The third form of job crafting relates to how employees change the cognitive boundaries of their work. Changing the cognitive boundaries involves employees altering their perceptions of their work, as either a set of discrete work tasks or as an important part of a larger whole. Changing their cognitive view of work in this way fundamentally influences how employees approach their work [17]. Thus, in contrast to relational and task crafting, cognitive crafting is a mental strategy and not a behavioural form of job crafting [17]. Cognitive crafting allows employees to be aware of the value they contribute through their work [18]. When employees either expand or limit the task, relational and cognitive boundaries of their work, they alter the design of the work and their social work environment. Such actions affect the meaning of the work to employees, i.e. ‘individuals’ understandings of the purpose of their work or what they believe is achieved in the work’, as well as their work identity, i.e. who they are at work [17]. Job crafting theory emphasises that employees utilise opportunities to actively design their work. It is thus characterised by a bottom-up process, rather than a top-down process where changes are designed and orchestrated by the organisation [17,19].

The theory proposes that through the use of job crafting strategies, employees can make adaptations to respond to the challenges posed by a job [20], such as the response to the COVID-19 pandemic. Job crafting may be a way for employees to handle demanding [19] and changing work situations [21,22] or a way of working through increased in uncertainty [23]. However, job crafting is not necessarily good for the employee. Changing the content of the work through job crafting requires energy, and if the individual’s ability to self-regulate is limited, job crafting may result in energy loss [24]. Moreover, job crafting may lead to an increase in burnout due to increased workload [25].

Healthcare professionals may use job crafting strategies in order to change aspects of their work [26]. Job crafting can thus be a powerful strategy through which healthcare professionals optimise their own performance at work (e.g. professional development) [26,27] and create a work situation that fits with their needs and preferences [26]. In the context of the COVID-19 pandemic, an earlier study shows that physicians felt challenged in their role as medical doctors and experienced that they were in a knowledge vacuum [28]. By applying job crafting theory, we can increase understanding of the action they took, the adaptations they made and how they crafted their work in this knowledge vacuum. We have not found any studies about job crafting by doctors in the context of COVID-19. We will therefore be able to add knowledge to current research about strategies used by GPs to deal with their situation at the beginning of the COVID-19 pandemic. Thus, the aim of this study is to identify how job crafting strategies were deployed by GPs at the beginning of the COVID-19 pandemic in Sweden and the significance of the strategies on their work situation.

## Methods

### Study design

A qualitative design has been applied for this study, using individual semi-structured interviews with GPs from different primary healthcare units in five Swedish regions, to gain a deeper understanding of GPs’ job crafting strategies.

### Setting

In Sweden, the majority of public healthcare services in hospitals and primary healthcare centres is provided through the country’s 21 self-governing regional authorities, referred to as regions [29]. The Swedish primary healthcare system, which is the setting of this study, is responsible for first-line healthcare [30], and provides care to members of the general public that do not require hospitalisation [31]. The responsibility for healthcare provision in Sweden is shared between the regions and local municipalities. Long-term care of elderly citizens, both care provided in nursing homes and home care support, is managed by the municipalities with the regions providing the municipalities with medical resources [32]. GPs thereby serve the citizens within the municipality-based elderly care system [33].

### Participants

GPs working within primary healthcare in Sweden were purposively recruited by the first and third authors. An invitation to participate in the study was advertised in social media and was distributed *via* e-mail by the Swedish District Medical Association in one region. Furthermore, the authors asked doctors in their networks to inform their colleagues and find out whether they were interested in participating. After each interview, participants were also requested to ask other GPs whether they would be interested in participating. The criteria for inclusion were that doctors had completed at least two of their five-year specialist-education in general medicine and were employed at a Swedish primary healthcare centre. Further criteria included having experience of working within primary healthcare at the beginning of COVID-19 pandemic. Fourteen primary healthcare doctors agreed to participate in this study and the GPs represented five Swedish regions. The characteristics of the study participants are listed in Table 1.

**Table 1.** Characteristics of study participants.

Characteristics	n
Sex	
Male	6
Female	8
Level of specialisation	
Specialist in general medicine	13
Resident in general medicine	1
Number of years after medical degree	
0–10	1
11–19	11
20+	2
Age	
<40	5
41–50	6
51–60	3

How many interviews that would be conducted to reach data saturation in this study was based on information power [34]. It has been suggested that the more information power a sample holds (i.e. information of relevance for the study), the fewer interviews are needed. The size of a sample with sufficient information power depends on the study aim, sample specificity, use of established theory, quality of the dialogue of the interviews and use of analysis strategy [34]. In this study sufficient information power was reached after fourteen interviews.

### Data collection

The interviews were conducted between June and November 2020. An interview guide was used containing a list of discussion themes to be covered with each participant. Discussion themes were derived from previous research into psychosocial working conditions, including the organisation of work, management, support, and changes in the healthcare system. The interview guide was tested in pilot interviews, and minor changes were made before the rest of the interviews were conducted.

The interviews were performed by two female researchers. Thirteen interviews were conducted by the first author (PhD student) and one by the third author (PhD). Eleven interviews were performed *via* the video platform Zoom, and three interviews *via* telephone. All participants agreed to their conversation being audio recorded. The interviews lasted 45–95 min with an average of 65 min. The technique of bracketing was applied throughout the research process [35] and to prevent personal interpretations during the interviews, the researcher ensured that the answers given by the GPs were correctly understood.

Only the participant and interviewer were present during the interviews to allow the participant to speak freely. None of the participants were previously known to either of the researchers performing the interviews. The audio-recorded interviews were transcribed verbatim by the first author immediately upon completion. Transcripts were labelled with a code, and information that could potentially have enabled the identification of the participant was removed. Furthermore, transcripts are stored on the authors’ password-protected computers and no unauthorised persons have access to the data.

### Data analysis

The interviews were analysed using qualitative content analysis, according to Graneheim and Lundman [36], with job crafting being the conceptual framework for the analysis process [17]. Three categories were thereby identified in advance: task crafting, relational crafting and cognitive crafting. Initially the first author read the transcripts several times to get an overall impression of the content. The NVivo qualitative analysis package (1.4.1) was used for the subsequent analysis. A categorisation matrix with the pre-defined categories of task crafting, relational crafting and cognitive crafting was created in NVivo. Thereafter, the text was condensed into smaller units of meaning and coded in line with the pre-defined categories [17]. The initial coding was done by the first author. Meaning units were coded under the task crafting category when the GPs discussed changes in the boundaries of their work tasks, for instance changing the type of work tasks. When GPs discussed their interactions with others at work, the meaning units were coded under the relational crafting category. When statements concerned GPs’ cognitive views of their work, and approach to the work, the meaning units were coded under the cognitive crafting category. The coded meaning units in each category were then grouped according to their meanings into sub-categories. Through the technique of bracketing [35], the analysis was carried out through continuous dialogue with all authors.

An example of the coding framework based on the job crafting categories is shown in Table 2.

**Table 2.** Coding framework example based on the job crafting categories.

Category	Sub-category	Code	Meaning unit
Task crafting	Take immediate action	Arranged equipment	‘I’ve been to Jula and Clas Ohlson [edit: retail stores], it’s been totally crazy, and bought loads of gear. I’ve bought a tent (…) I’ve been a bit impressed, and thought, what a huge thing we’ve achieved.’ (GP 14)
Relational crafting	Social relations support	Discuss approaches to clinical work	‘And then we discussed together. What do you think? What should I think? What should I do with patients who come in with a really high pulse and normal breathing? How should I act? And they complain that they have trouble breathing. We discussed like that back and forth.’ (GP 10)
Cognitive crafting	Strengthened work identity	Proud to contribute	‘It was sad not getting any support, but I’m really proud to feel I have done everything I can from my perspective as a doctor.’ (GP 4)

## Ethical considerations

Ethical application was approved by the Swedish Ethical Review Authority (2020-02433). All data was handled and stored in accordance with the Swedish Act on Ethical Review of Research Involving Humans [SFS 2003:460 (2005)].

Before the interviews all participants were given information about the study, their rights and that they could withdraw their participation whenever they wanted. Informed written consent to participate was obtained *via* e-mail. Participants were assured of confidentiality and that their full identity would not be known to anyone but the researchers. No participant withdrew their participation during or after the interviews.

## Results

In their endeavours to organise and provide care during the response to the COVID-19 pandemic, GPs shaped the task, relational and cognitive boundaries of their work. Within the three main job crafting categories, the analysis of the data yielded three sub-categories, one in each main category. These sub-categories describe how the job crafting strategies were deployed and the significance of the strategies to the GPs’ work situation.

### Task crafting

The task crafting category is concerned with how the GPs changed the type of work tasks, and from the interviews it emerged that GPs used different task crafting strategies. These strategies were characterised by immediate action with GPs rapidly managing problems as they arose in order to organise and provide care.

### Take immediate action

GPs experienced that their work tasks were given lower priority than tasks performed within hospital-based care. The information provided by the higher levels of the healthcare organisation was largely focused on the management of COVID-19 within hospital-based care, and was less clear regarding how care should be provided within primary healthcare. GPs described that it was up to each unit to decide how it would act to provide care and prevent the spread of the virus. This meant that GPs and their colleagues in the unit felt that they had plenty of room to manoeuvre in the organisation of care.

Once again, there hasn’t really been much support centrally (…), and we’ve had to solve things ourselves at the healthcare centre instead. (GP 12)

Instead of remaining passive, GPs reported that they shaped their work tasks, and they proved their ability to structure the organisation and provision of care. Shaping their work tasks gave GPs a sense of pride in their ability to find new ways of working.

It was suddenly possible to influence quite a lot, fairly concrete things (…) we can really turn the organisation upside down and still make it work. We completely changed how we worked from one day to the next. (GP 2)

GPs expressed that they managed problems as they arose by shaping their clinical work tasks. Through taking immediate action, GPs and their colleagues in the unit experienced that they solved practical matters related to the organisation of care and helped to limit the spread of the virus. From one day to the next, GPs reorganised the work environment within their units. They described that they and their colleagues established separate entrances or external facilities such as tents so they could examine patients who had infectious symptoms. There were even some instances when GPs were involved in buying these tents and other materials needed to limit the spread of the virus.

I’ve been to Jula and Clas Ohlson [edit: retail stores], it’s been totally crazy, and bought loads of gear. I’ve bought a tent (…) I’ve been a bit impressed, and thought, what a huge thing we’ve achieved. (GP 14)

Some GPs said that they were critical of the lack of PPE within municipal elderly care. To limit the spread of the virus and protect frail elderly citizens, they arranged PPE themselves and gave it to the staff within elderly care. These actions were taken without the knowledge of their superiors as they had been told that PPE within elderly care was not a priority at that time. GPs described that in their clinical work, they developed new ways to interact with their patients without physical meetings, such as switching to digital solutions, *via* telephone or video.

It’s doable when things are really critical, and it was suddenly possible to arrange and start with video appointments in no time. (GP 11)

However, some patients still needed to go to the primary healthcare centre and GPs described that they acted by carefully considering which patients to prioritise for physical consultations. In other words, they assessed the risk versus the benefit of the physical visit. GPs reported that to facilitate their clinical work and care for the patients, they sometimes defined their own routines along with other GPs within their units. For instance, they decided how to prioritise physical patient visits, and also how they should deal with and prioritise frail elderly people and those with chronic diseases. GPs responsible for municipal elderly care expressed that their actions included more frequent conversations with the elderly about the level of care if they were to get sick, as well as making documented plans about treatment strategies for these patients, for example, if hospital care was relevant.

### Relational crafting

The relational crafting category covers how GPs describe their interaction with others, and the quality of those interactions. GPs expressed that their work situation allowed them room to manoeuvre, which enabled them to shape their relational boundaries and continuously seek support from other healthcare professionals.

## Social relations support

GPs described that due to a feeling of uncertainty and the lack of clarity about how primary healthcare professionals should act and solve matters, they interacted in new ways with others to get information and support. Some GPs expressed a strengthened feeling of solidarity with other healthcare professionals. GPs explained that shaping their relational boundaries made them more confident in their clinical work and gave them a sense of community of practice. They described intensified collaboration with other GPs within their units, including continuously updating each other on the organisation of work and listening to each other’s opinions. Further, GPs described that the uncertainty regarding COVID-19 management strategies raised many questions, which were dealt with through collegial discussions.

What’s happened since yesterday? Or maybe, what shall we do with this now? I know we were supposed to change. How are things now? So there’s really been a lot of communication, and good communication I believe (…). How do you do it? What do you think about this personal protective equipment? Or whatever question it may be. (GP 1)

And then we discussed together. What do you think? What should I think? What should I do with patients who come in with a really high pulse and normal breathing? How should I act? And they complain that they have trouble breathing. We discussed like this back and forth. (GP 10)

Discussing with other GPs was also described as a strategy to manage more concerning emotions. For example, when GPs questioned themselves in their medical decisions and also experienced fear that their patients would become seriously ill or die.

That I have somebody I can discuss with who understands how I think (…). And I also had a great deal of support from a doctor colleague. So then I felt that, oh, there is someone else who understands and someone who thinks like me, and that relieves the stress enormously. (GP 8)

Furthermore, GPs described that they collaborated with GPs from other primary healthcare units through which they validated their questions and concerns, for instance regarding how other units were acting, made medical decisions, and arranged practical matters related to COVID-19 care.

We networked a little with other healthcare centres (…). Where did you buy this tent? What size did you get? How did you fix it in the ground? Those kinds of practical questions. And how do you deal with chronically ill people? (…). We had good dialogue within the region between doctors, and healthcare centres. And this has made us stronger. (GP 14)

Over and above the collaboration between GPs, they described that cohesion started to develop across professional boundaries, e.g. with nurses and assistant nurses. One far from insignificant example was the intensified collaboration with nurses within municipal elderly care, which was described as being necessary to provide care of the elderly, and reasoning about the management of COVID-19 cases in municipal elderly care. GPs said that the interactions with municipal nurses sometimes took place on a daily basis, which not was the case before the onset of the pandemic.

Another difference is that I’ve probably worked more closely with the municipal nurses in a way (…). More time for discussions if I were to think of one difference from before (…). There’s more, closer collaboration, we talk through how we’re going to deal with things. (GP 2)

When concerns regarding medical assessments could not be solved with the existing competence within primary healthcare, GPs described that they contacted specialist healthcare professionals and acted based on their recommendations. One example of this was when uncertainty prevailed regarding what to do in the event of a virus outbreak at municipal nursing homes.

And I also had help from Infectious Diseases at the hospital, I phoned them a few times (…). I always knew who to ask if I didn’t know something. (GP 7)

The support that GPs sought from colleagues was not only face-to-face or verbal. GPs described that they identified strategies for receiving support *via* social media, and that they discussed COVID-19-related issues in Facebook groups for doctors. These included treatment strategies, how symptoms of COVID-19 should be interpreted, and how to solve problems regarding personal protective equipment.

We constantly received information from the region, but I realised that it wasn’t enough. I need more to be able to do my job (…) There was loads of information about how they should deal with patients in hospitals, but not that much about what we were meant to do in primary healthcare. What’s expected of us? This was lacking for me, so I looked for it in other media, such as Facebook. (GP 8)

### Cognitive crafting

The cognitive crafting category concerns GPs’ cognitive view of and approach to their work. From the interviews it emerged that GPs used cognitive crafting strategies, which was reflected in a strengthened work identity.

## Strengthened work identity

Even though the GPs interviewed expressed that management of COVID-19 within primary healthcare was given lower priority and limited support from the higher organisational levels, they describe their independence in a more favourable light. In other words, GPs described that they took charge over how care would be organised and implemented. Some GPs described that doctors became the ‘new leaders’ within the healthcare organisation. GPs expressed that their readiness for action and ability to solve matters on their own gave them increased self-confidence in their profession.

It was sad not getting any support, but I’m really proud to feel I have done everything I can from my perspective as a doctor. (GP 4)

The experience of working as a doctor during the pandemic was described as challenging but also stimulating. Some GPs viewed this experience and the lessons learned as invaluable, and an important experience for their future professional life. During this time, GPs developed their clinical skills, and became stronger at making sometimes difficult medical decisions. For example, GPs described an improved ability to see when their patients were seriously ill with COVID-19. To limit the spread of the virus, GPs were more limited in their use of physical examinations of patients and examinations using diagnostic aids (e.g. x-ray). They described that this more restrictive approach helped develop their clinical skills.

So I kind of had to work more with intuition and more with reasoning back and forth with myself and with the patient. Shall I give you antibiotics or shall we wait? So I was forced to become a stronger clinician (…). Left more to my own professional decision making, you know, and that was great. (GP 9)

GPs described that their view of and approach to their work strengthened their work identity. Put another way, GPs’ ability to shape their clinical work tasks in the organisation of care and development of clinical skills, as well as their sense of importance of being a part of a larger whole, was enhanced. GPs expressed that they felt strengthened when they viewed themselves from a wider societal perspective as a part of fighting the pandemic more broadly. They described being involved in this work as significant, doing what they could to help those who needed help, and supporting the maintenance of a functioning society.

Actually working with something where you can help, be involved and take care of what was going on in some way, even if it’s just my tiny, tiny part of it all, but nonetheless. (GP 11)

## Discussion

The aim of this study was to identify how job crafting strategies were deployed by GPs during the COVID-19 pandemic in Sweden and the significance the strategies had on their work situation. We used job crafting theory to explore how GPs actively used task, relational and cognitive crafting strategies to deal with their work situation in response to the COVID-19 pandemic. The job crafting strategies used in response to COVID-19 were characterised by having room for manoeuvre, which is reflected in the GPs’ abilities to take immediate action, their collaboration with other healthcare professionals and their strengthened work identity.

Existing literature describes that job crafting involves the shaping of the job by the employee and not by the organisation [17,19]. Similarly, our study found that many of the changes in the clinical work tasks of GPs during the response to the pandemic were initiated by the GPs themselves. When employees are given less attention from management in their work, they can identify more opportunities to use job crafting strategies [17]. In our study, GPs experienced that they had limited attention from the higher levels of the organisation, and that it became incumbent on GPs and their unit colleagues to decide how they should act to organise care. GPs shaped their task boundaries [17], and the room for manoeuvre they had meant that they performed work tasks they normally did not do. Consistent with previous studies carried out during the COVID-19 pandemic [7,11], GPs proved their ability to improvise and use creative strategies. GPs in our study felt that they managed problems as they arose and tried to fight the pandemic to the best of their ability. They had a high degree of involvement in transitioning and adapting primary healthcare to the COVID-19 pandemic and in the development of strategies for the continued care to the patients.

Nevertheless, GPs described that they could not handle the uncertainty the pandemic generated on their own. Their work situation made it possible for them to shape the relational boundaries of their work [17]. One way they altered the relational boundaries was by establishing contact with colleagues to get work-related information [37]. Similar to previous studies [7,10,12–14,16], the GPs interviewed experienced that the pandemic brought about more intense collaboration with colleagues in order to handle their day-to-day tasks. This collaboration was valuable for information exchange and for discussing the uncertainty that GPs felt in medical decisions and other practical matters [7,14]. As shown in another Swedish study [12], GPs in this study described more extensive cooperation with other units, and in particular a closer relationship with nurses within the municipality-based care. This collaboration was described as important for ensuring the care of the elderly. As a consequence, collaboration with other healthcare professionals created a sense of community of practice among GPs. Relational crafting can thus help employees to build social relationships and positive interactions with others [38].

Literature on job crafting highlights the relationship between cognitive crafting and work identity [17]. This is confirmed in our study, which showed that working during the pandemic affected GPs’ cognitive view of their own work role. The GPs’ work identity was thereby strengthened by their own view of their ability to organise and implement care. It has been shown that cognitive crafting can promote employees’ awareness about their contribution to work [18]. In our study, GPs describe how they took charge of how care would be organised and implemented. Consistent with previous research [11], some GPs stated that during the COVID-19 pandemic they became the ‘new leaders’ within the healthcare organisation. Cognitive crafting can involve viewing work tasks as an important part of a larger whole, which also influences how employees approach their jobs [17]. This aspect of cognitive crafting is also found in our results where GPs, over and above their desire to do good for their patients, expressed that they also wanted to do good for the wider society. This cognitive view of their work in turn led to a strengthened work identity.

Even though job crafting is considered to be a voluntary action [17], our results indicate that making changes in the work situation was a necessity in GPs’ efforts to continue to provide care.

However, the conditions of the work situation had an impact on how GPs chose to job craft. The ample room for manoeuvre created during the response to the pandemic gave GPs the opportunity to change the ways of working in relation to the organisation and provision of care, and to seek support from other healthcare professionals. In addition to the provision of care, the changes GPs made in the task, relational and cognitive boundaries of their work were experienced as bringing additional meaning [17] to their clinical practice. The GPs expressed that the ability to organise and provide care gave them a sense of pride, while collaboration with other healthcare professionals made GPs more confident in their clinical work and gave them a sense of community of practice. Furthermore, GPs experienced increased self-confidence and were strengthened in their professional role due to their accomplishments in relation to the organisation and implementation of care.

Previous research shows associations between job crafting and well-being among healthcare professionals [39]. However, the question remains whether the job crafting strategies used by the GPs interviewed in response to the pandemic are sustainable over time. The use of job crafting strategies can act as a short-term solution during a demanding period [19]. In addition, job crafting requires energy and if drained over time it may result in fatigue [24] and burnout [25]. A Swedish study found exhaustion among 16.7% of GPs during the COVID-19 pandemic [40]. This may thus indicate that even though GPs crafted their jobs to handle their situation during the pandemic, they might still suffer from poor mental health.

### Methodological considerations

There are a number of limitations which should be taken into consideration in interpreting the results of this study. The transferability of the results is limited to the Swedish primary healthcare setting, and the results cannot directly be transferred to an international setting.

The questions in the interview guide took the perspective of psychosocial working conditions during the pandemic, such as organisation of work, management, support, and changes in the healthcare system. As such, the questions were not specifically based on job crafting, which can be considered a limitation. However, during the analysis process GPs’ strategies for dealing with clinical work tasks appeared clearly. Viewing GPs’ strategies from a job crafting perspective could add value in the analysis. There are also considerable strengths to the study. One strength of the study is that sufficient information power was reached [34], and based on that, GPs were very articulate and provided information-rich material. All the GPs in our study had experience of working as doctors in primary healthcare during the beginning of the COVID-19 pandemic. Furthermore, the aim of the study was relatively narrow, aiming to present selected patterns relevant to the study aim, and not to cover the whole range of phenomena. The study was also supported by established theory. After fourteen interviews the data collection was considered sufficient as no significant new information emerged in the last interviews. According to Guest et al. [41], twelve interviews should be sufficient to reach data saturation if the above mentioned criteria of Malterud et al. [34] are considered fulfilled.

The authors behind the study consisted of a registered physiotherapist, a public health scientist, a registered nurse, and a social scientist.

Three of the authors bring valuable prior experience from their work in healthcare settings. Also, three authors possess extensive expertise in studying healthcare professionals and are experienced in qualitative research. This calls for reflexivity through the research process i.e. recognizing the influence of preconceptions especially during the interviews and analysis [42]. Employing the technique of bracketing becomes crucial to mitigate potential adverse effects stemming from preconceptions [35]. For instance, during the interview phase, the researcher carefully reflected on the responses provided by GPs to prevent personal interpretations and avoid making a priori assumptions about their intended meaning. Further, to enhance trustworthiness [36], the authors meet regularly to reflect upon the analysis of interviews, discussing the coding process, the development of sub-categories in relation to theoretical categories until a consensus was reached. This iterative process not only enhances the rigor of the study but also ensures a comprehensive and well-grounded interpretation of the gathered data.

The participants did not give feedback during the analysis in order to seek agreement [36]. However, the participants were informed about the availability of the final results. The study has been reported according to the COREQ 32-item checklist for qualitative studies [43].

## Conclusions and implications

The results of this study provide insight into how GPs deployed job crafting strategies and the significance the strategies had on their work situation when facing the COVID-19 pandemic.

The task, relational and cognitive job crafting strategies GPs used had meaning to them in clinical practice. The conditions of the work situation enabled substantial room for manoeuvre, through which GPs felt proud in finding new ways of working. GPs described that collaboration between healthcare professionals helped them become more confident in their clinical work and gave them a sense of community of practice. Consequently, GPs experienced that their professional role was strengthened through what they accomplished in relation to the organisation and implementation of care. This study explored GPs’ job crafting strategies in the context of the COVID-19 pandemic, and the question remains as so whether GPs job crafted their work tasks due to the demanding conditions that prevailed. It is also unknown whether job crafting affects GPs’ mental health over time. Future studies should investigate the long-term effects of job crafting behaviour on GPs, and its impact on their mental health. Our study highlights GPs, a group of doctors that received less attention when dealing with the COVID-19 pandemic. The knowledge about how GPs used job crafting strategies, and the meaning of these strategies in clinical practice, might be useful for healthcare organisations in preparing for future health crises. Taking advantage of GPs’ experiences and strategies is considered important for promoting sustainable working conditions for GPs in the future.

## Data Availability

The datasets generated and analysed during the current study are not publicly available. Participants have consented to the publication of aggregated data, but not to open publication of data for individuals. Data is available from the corresponding author upon reasonable request.
